# Therapeutic Potential of Mesenchymal Stem Cell-Secreted Factors on Delay in Corneal Wound Healing by Nitrogen Mustard

**DOI:** 10.3390/ijms231911510

**Published:** 2022-09-29

**Authors:** Seungwon An, Xiang Shen, Khandaker Anwar, Mohammadjavad Ashraf, Hyungjo Lee, Raghuram Koganti, Mahmood Ghassemi, Ali R. Djalilian

**Affiliations:** 1 Department of Ophthalmology and Visual Sciences, University of Illinois at Chicago, Chicago, IL 60612, USA; 2 Department of Pathology, Shiraz University of Medical Sciences, Shiraz 71348-14336, Iran

**Keywords:** nitrogen mustard, mesenchymal stem cell-conditioned media, corneal injury, apoptotic cell death, cellular ROS, delayed wound healing

## Abstract

Ocular surface exposure to nitrogen mustard (NM) leads to severe ocular toxicity which includes the separation of epithelial and stromal layers, loss of endothelial cells, cell death, and severe loss of tissue function. No definitive treatment for mustard gas-induced ocular surface disorders is currently available. The research was conducted to investigate the therapeutic potential of mesenchymal stem cell-conditioned media (MSC-CM) in NM-induced corneal wounds. NM was added to different types of corneal cells, the ocular surface of porcine, and the ocular surface of mice, followed by MSC-CM treatment. NM significantly induced apoptotic cell death, cellular ROS (Reactive oxygen species), and reduced cell viability, metabolic gene expression, and mitochondrial function, and, in turn, delayed wound healing. The application of MSC-CM post NM exposure partially restored mitochondrial function and decreased intracellular ROS generation which promoted cell survival. MSC-CM therapy enhanced wound healing process. MSC-CM inhibited NM-induced apoptotic cell death in murine and porcine corneal tissue. The application of MSC-CM following a chemical insult led to significant improvements in the preservation of corneal structure and wound healing. In vitro, ex vivo, and in vivo results suggest that MSC-CM can potentially provide targeted therapy for the treatment of chemical eye injuries, including mustard gas keratopathy (MGK) which presents with significant loss of vision alongside numerous corneal pathologies.

## 1. Introduction

Nitrogen mustard (NM) is a bifunctional alkylating analog of the chemical warfare agent sulfur mustard (SM). NM and SM exposure to the eye cause severe ocular toxicity, abnormal corneal structure, and separation of epithelial and stromal layers [1,2,3]. NM directly reacts with the thiol or amino group of proteins to induce DNA-protein crosslinking which can lead to mutagenic and cytotoxic effects [4]. Indirectly, NM targets DNA repair replication endonucleases, resulting in single-strand breaks (SSBs) and double-strand breaks (DSBs) in DNA [5,6]. DSBs, in particular, cause cell cycle arrest, genome stability, and chromosomal damage [7]. NM exposure in ex vivo and in vivo rabbit corneal injury models promoted apoptosis, epithelial–stromal separation, corneal collagen degradation, and neovascularization [3,5,8,9,10]. Earlier studies using the murine skin injury model and rabbit corneal culture model demonstrated that exposure to the vesicating chemical agents sulfur mustard (SM) and nitrogen mustard (NM) exposure led to increases in apoptotic cell death and epidermal thickness and biomarkers, such as COX2 (cyclooxygenase 2, mediation of inflammation), MMP-9 (matrix metalloproteinase-9, inflammatory marker), and VEGF (vascular endothelial growth factor, angiogenic factor) [11,12,13]. NM and SM exposure also increased oxidative stress and oxidative stress-induced pathways may prevent by antioxidant treatments [3,14,15].

Studies have demonstrated the therapeutic efficacy of dexamethasone, doxycycline, silibinin, and human fibroblast growth factor-1 derivative in ameliorating NM-induced corneal injuries [13,16]. However, NM exposure can develop into a chronic or delayed-onset mustard gas keratopathy (MGK) which presents with significant loss of vision alongside numerous corneal pathologies [17,18,19,20,21,22]. The list of MGK complications is extensive and includes primary changes such as corneal opacity, neovascularization, epithelial defects, scarring, chronic blepharitis, meibomian gland dysfunction, dry eye, limbal ischemia, stem cell deficiency, and secondary degenerative changes, including lipoid deposits and amyloid deposition [17,18,19,20,21,22]. No definitive treatment for chronic and delayed mustard gas-induced ocular surface disorders is currently available. The pathogenesis of MGK is thought to be mediated in part by oxidative stress induced by reactive oxygen species [23]. Oxidative stress can induce lipid, protein, and DNA oxidation, which can modify the functional activity of cellular components and ultimately impair viability [24,25]. For mustard toxicity, rats were administrated thiosulfate, vitamin E, or dexamethasone, and it showed increases in survival and fewer pathological organ effects [26]. The topical cortisone treatment also decreased NM-induced edema which caused less deep skin lesion in rabbits [27].

Mesenchymal stem cells (MSC) have been extensively studied for their therapeutic usage due to their anti-inflammatory and immunomodulatory effects [28,29]. MSCs have also been shown to have significant antioxidative properties. Injection of MSCs protects against ischemia–reperfusion-induced vascular damage, hypoxia, oxidative DNA damage, and apoptosis in vivo [30]. Similarly, MSC-conditioned media (MSC-CM) has been shown to inhibit H_2_O_2_-induced ROS accumulation which suppressed cell death [30]. MSC-CM protects oxygen–glucose-deprivation-induced spinal cord glial cell damage in vitro and improved the functional capabilities of rats after spinal cord injury in vivo [31,32,33,34]. In ocular tissues, MSC-CM has anti-inflammatory and anti-fibrotic effects during corneal repair [35]. Furthermore, our previous study reported that lyophilized conditioned media from corneal MSCs stimulate corneal epithelial wound healing [36].

MSCs may present a promising therapeutic avenue for the treatment of MGK due to their anti-inflammatory and anti-oxidative effects. This study first examined the dose response of NM on freshly isolated murine and porcine corneas and later explored the protective effects of MSCs-secreted factors after NM exposure in in vitro, ex vivo, and in vivo models. We demonstrated that NM exposure significantly induced corneal epithelial and stromal damage in part by inducing mitochondrial membrane changes and increasing cellular ROS. MSC-CM attenuated the NM-mediated induction of cellular ROS which resulted in the preservation of the corneal epithelium–stroma structure ex vivo. Finally, MSC-CM accelerated corneal wound healing in vivo. Therefore, MSC-CM may be beneficial in the development of targeted therapeutics against NM-induced ocular injury.

## 2. Results

### 2.1. Effects of Nitrogen Mustard on Viability In Vitro

**Figure 1:**
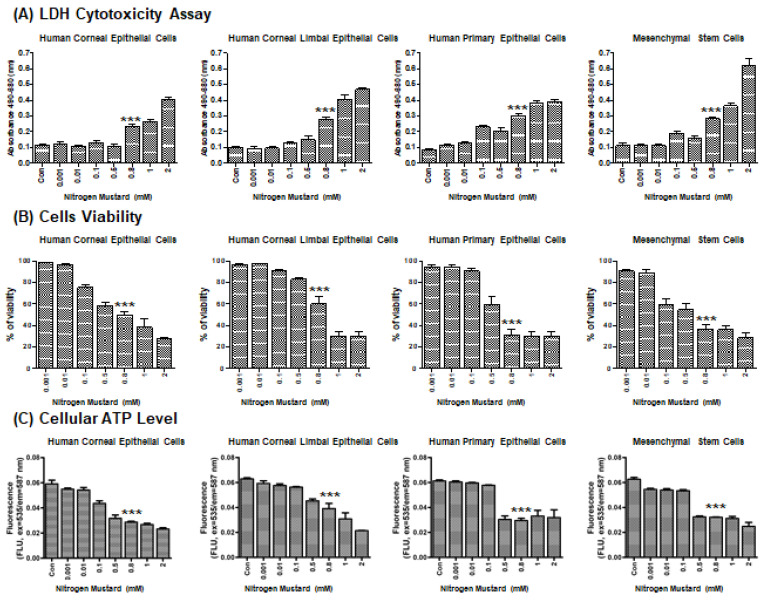
Effects of nitrogen mustard exposure on cell cytotoxicity, cell viability, and ATP level in culture cells. (**A**) Graph showing cytotoxicity for various doses of nitrogen mustard (NM) in culture cells as measured by LDH assay (n = 6/group). (**B**) Graph showing cell viability for various doses of NM in culture cells as measured by LDH assay (n = 6/group). (**C**) Graph showing ATP levels for various doses of NM in culture cells as measured by LDH assay (n = 6/group). *** *p* < 0.001 vs. untreated control or 0.001 mM NM treated group.

To evaluate NM-mediated cell injury on the ocular surface, we performed a lactate dehydrogenase (LDH) cytotoxicity assay to determine the 50% cytotoxicity concentration (CC50) of NM in the following cell types: human corneal epithelial cells (HCECs), human corneal–limbal epithelial cells (HCLE), human primary corneal epithelial cells (HCEpCs), and human cornea MSCs (Figure 1A). NM exposure resulted in a dose-dependent decrease in cell viability beginning at 0.1 mM NM in HCECs, HCLEs, and MSCs (Figure 1B). HCEpCs began to experience toxic effects at 0.5 mM NM (Figure 1B). The CC50 of NM was approximately 0.8 mM in the four cell types (Figure 1B). In parallel experiments, the cellular ATP levels were measured in each cell type with increasing dosages of NM. Here, too, NM caused dose-dependent decreases in ATP levels in all cell types (Figure 1C). These experiments established that 0.8 mM NM induces approximately 50% loss of viability in each cell type, and thus we used this dose to establish our model of NM-mediated cell injury.

### 2.2. MSC-CM Rescues Immediate Cytotoxic Effects and Enhances Wound Healing in Corneal Epithelial Cells Exposed to NM

**Figure 2:**
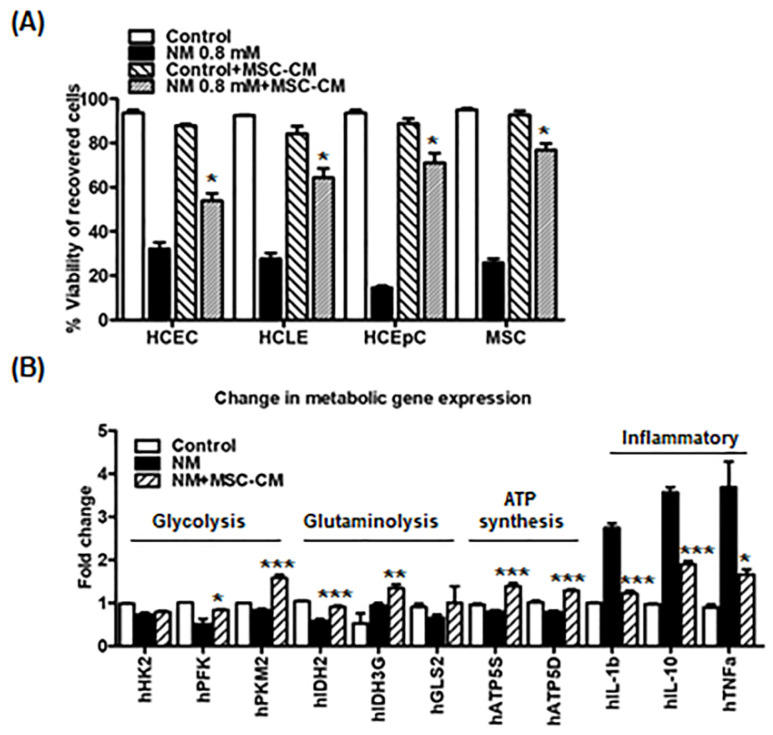
Mesenchymal Stromal Cell (MSCs)-conditioned media recovers immediate cytotoxic effects in the corneal epithelial cells following exposure to nitrogen. (**A**) Cell viability of recovered cells is measured by trypan blue staining with control, NM 0.8 mM, control media + MSC-CM, and NM 0.8 mM + MSC-CM. *** *p* < 0.001 vs. NM 0.8 mM. (**B**) Graph showing the mRNA levels of genes for glycolysis, glutaminolysis, ATP synthesis, ATP synthesis, and inflammatory genes in control, NM 0.8 mM (2 h), and NM 0.8 mM (2 h) + MSC-CM (24 h) by performing qPCR. HCEC cells are used for NM treatment. * *p* < 0.05, ** *p* < 0.01, *** *p* < 0.001 vs. NM treated group.

We then examined the effects of MSC-CM on cell viability after NM exposure. As noted above, 0.8 mM NM exposure for 2 h induced significant cell death in all cell types (average of cell viability; HCEC: 32.1 ± 6.4%, HCLE: 27.6 ± 5.8%, HCEpC: 14.6 ± 1.6%, and MSCs: 25.7 ± 4.5%) (Figure 2A). Incubation with MSC-CM post 0.8 mM NM exposure improved cell survival significantly compared with the NM treatment group (average of cell viability; HCEC: 53.9 ± 7.4%, HCLE: 64.2 ± 9.7%, HCEpC: 71.0 ± 9.8%, and MSCs: 76.7 ± 6.6%) (Figure 2A). Next, we investigated the effects of MSC-CM on metabolic gene expression in epithelial cells following exposure to NM. As anticipated, NM exposure significantly decreased metabolic gene expression (glycolysis, glutaminolysis, and ATP synthesis) compared with the control group (Figure 2B). Inflammatory genes were also significantly increased following NM exposure (Figure 2B). However, the changes in gene expression were prevented by the addition of MSC-CM following NM exposure (Figure 2B). These observations indicate that NM exposure down-regulated metabolic gene expression, whereas MSC-CM partially rescued NM-mediated cell damage.

### 2.3. MSC-CM Attenuates NM-Induced Change in Mitochondrial Membrane Potential

**Figure 3:**
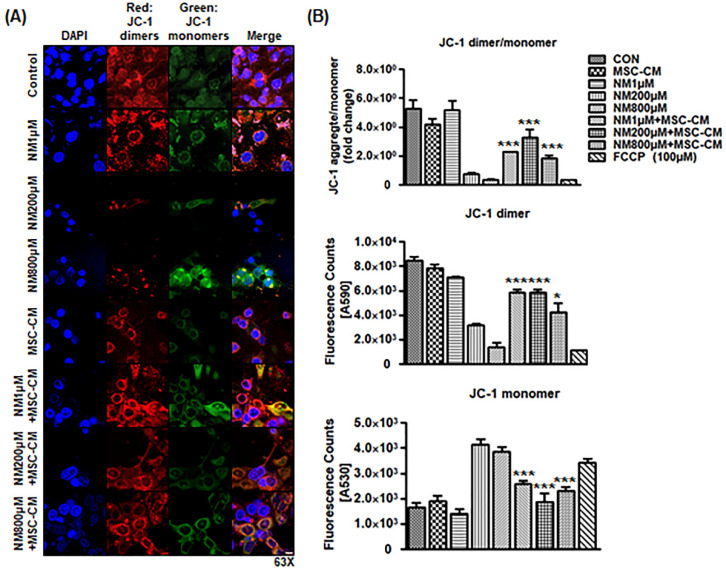
MSC-CM attenuates NM-exposed mitochondrial membrane potential change. (**A**) Representative brightfield images after NM exposure or NM with MSC-CM incubation. Representative fluorescence microscopy images of JC-1 dimers (red) and JC-1 monomers (green) after various doses of NM with or without MSC-CM. The merged panel presents co-localization between red dimer and green monomer fluorescence. Scale bar, 10 μm (**B**) Fluorescence intensity of mitochondria membrane potentials (JC-1 dimer, JC-1 monomers, and JC-1 dimer/monomer) by fluorimetry. FCCP 100 μM was used as depolarization negative control. * *p* < 0.05, *** *p* < 0.001 vs. NM treated group.

On the basis of the changes in metabolic gene expression profile following MSC-CM treatment, we hypothesized that MSC-CM prevents NM-induced mitochondria dysfunction in corneal cells. To test this hypothesis, confocal microscopy was used to detect J-dimers and J-monomers in the mitochondria of HCEC cells following incubation with NM or NM+MSC-CM for 3 h. JC-1 staining (red color: dimers, high potential; green color: monomers, low potential) showed that control and NM 1 μM treatment still maintained high mitochondrial membrane potential (MMP, JC-1 dimer/monomer ratio), whereas following NM exposure, levels of the JC-1 monomers (green) were increased and JC-1 dimers (red) were decreased, whereas the NM + MSC-CM combination group maintained higher JC-1 dimers and lower JC-1 monomers. These results showed a significant increase in the red/green fluorescence ratio in NM + MSC-CM combination compared with NM exposure group, indicating that the NM depolarized the MMP of the HCEC cells, whereas MSC-CM incubation repolarized the MMP afterward (Figure 3A). As the MMP is a key indicator of mitochondrial activity, MSC-CM appears to preserve mitochondrial function after NM exposure. In a parallel experiment, JC-1 fluorescence intensity was measured after three dosages of NM (1, 200, and 800 μM) with or without MSC-CM in epithelial cell culture. NM exposure decreased the ratio of JC-1 dimers/monomers, whereas MSCs-secreted factors reversed the NM-mediated drop in the JC-1 ratio (Figure 3B). Collectively, these results suggest that mitochondrial function was impaired by NM exposure and restored by MSC-CM therapy.

### 2.4. MSC-CM Decreases Cellular ROS Generation

**Figure 4:**
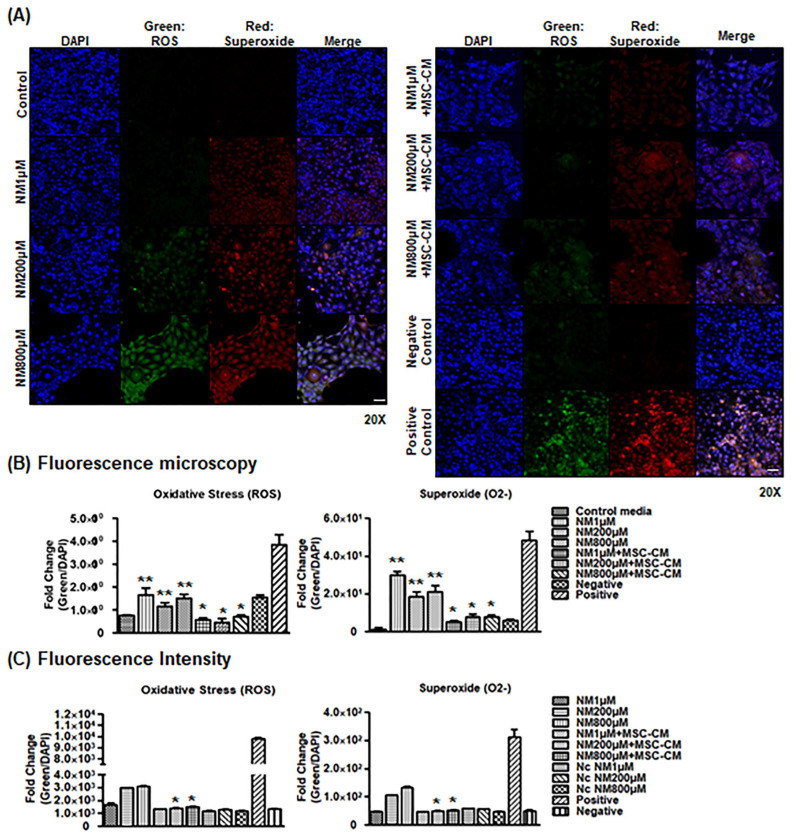
MSC-CM decreases cellular ROS generation. (**A**) Representative confocal immunofluorescence microscopy images of ROS (green) and Superoxide (red) after various doses of NM with or without MSC-CM. Scale bar, 50 μm (**B**) Relative fold changes from Figure 4A. ** *p* < 0.01 vs. Control media, * *p* < 0.05 vs. NM treated group. (**C**) Representative ROS/Superoxide assay showing the effect of MSC-CM on NM exposure in cell culture. Fluorescence intensity after NM (1, 200, and 800 uM) and NM with MSC-CM incubation for 2 h. Positive control (ROS Inducer: Pyocyanin). Negative Control (ROS Inhibitor: N-acetyl-L-cysteine). Negative Control (solvent). * *p* < 0.05 vs. NM treated group.

Disruption of mitochondrial dynamics is associated with an increase in mitochondrial oxidative stress and ROS generation. In parallel in vitro experiments, cellular ROS was measured to determine the effect of MSC-CM after NM exposure on epithelial cells. Fluorescence microscopy images of ROS and superoxide (O_2_^−^) generation after incubation with NM or NM + MSC-CM showed that NM treatment increased ROS and superoxide accumulation in a NM dose-dependent manner (Figure 4A). ROS and superoxide were also co-localized in epithelial cells. However, MSC-CM incubation after NM exposure decreased ROS and superoxide detection in epithelial cells compared with NM alone (Figure 4A,B). To determine whether NM exposure can induce cellular ROS generation in vitro using ROS/superoxide fluorescence intensity, we treated epithelial cells with NM alone or NM with MSC-CM. As shown in Figure 4C, NM exposure increased ROS accumulation in a dose-dependent manner, while MSC-CM treatment after NM exposure significantly decreased NM-induced cellular ROS and superoxide generation (Figure 4C). Therefore, NM exposure damages mitochondria and increases oxidative stress [37,38,39], while MSC-CM functions as an antioxidant to combat these deleterious effects.

### 2.5. Effect of MSC-CM on Murine and Porcine Corneal Wound Healing after NM Injury

**Figure 5:**
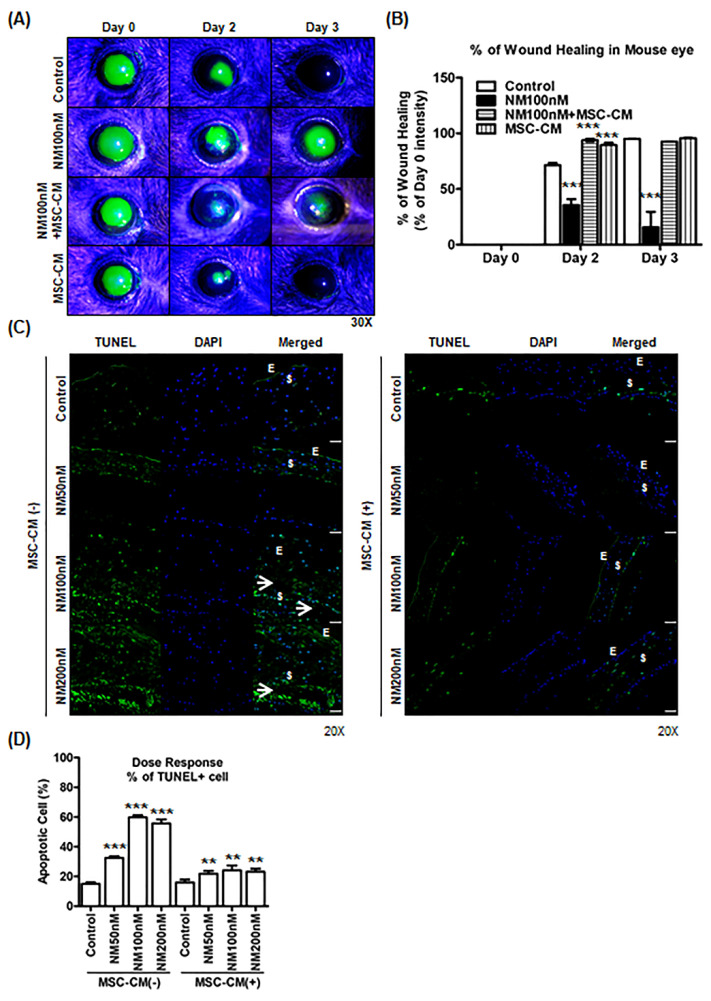
Wound healing effect of MSC-CM on NM injury on mouse corneas. (**A**) Representative images of mouse corneas (n = 4/group) showing fluorescein staining after epithelial scratch and topical application of 10 μL of NM 100 nM on the center of cornea for 30 min. After 30 min, corneas were washed with 1 mL of 1X PBS, and corneas were applied with MSC-CM twice a day for 3 days. (**B**) Relative fold changes of comparing epithelial defect area between groups at Day 0 to Day 3 (n = 4/group) from Figure 5A. *** *p* < 0.001 vs. untreated control (Day 2) or untreated control (Day 3). (**C**) Ex vivo mouse eye culture model: For apoptotic cell death, mouse eyes were cultured in various doses of NM (50, 100, and 200 nM) in KSFM media for 2 h and washed two times with 1X PBS. After washing, NM-exposed mouse eyes were replaced with (**B**) or without (**A**) MSC-CM for 2 days and imaged using 20× magnification im-immunofluorescence by TUNEL assay. TUNEL (Green), DAPI (Blue). Scale bar, 50 μm (**D**) Graphs showing apoptotic cell death on mouse cornea area from Figure 5C. *** *p* < 0.001 vs. untreated control (MSC-CM-), ** *p* < 0.01 vs. NM treated group (MSC-CM-).

After establishing the capacity of MSC-CM to treat NM injury in vitro, we performed experiments to determine whether MSC-CM can promote corneal epithelial healing after NM injury in vivo. Mice were subjected to a corneal epithelial scratch followed by 100 nM NM or NM+MSC-CM. Wound healing was significantly reduced after NM exposure compared with the control cornea (41.03 ± 45.02%), but the mice given MSC-CM after NM exhibited near complete healing of their epithelium after Day 3 post-wounding (Figure 5A,B). It suggested that NM exposure delayed corneal wound healing after mechanical wound compared with MSC-CM treatment group. Next, we investigated the effects of MSC-CM on NM-induced apoptotic cell death in an ex vivo murine eye model. Exposure of 50–200 nM NM resulted in significant increases in apoptosis of the murine corneal tissue compared with control (Control, 15.0 ± 2.4%; NM 50 nM, 32.5 ± 3.2%; NM 100 nM, 59.8 ± 3.8%; and NM 200 nM, 54.7 ± 7.8%) (Figure 5C). However, MSC-CM incubation significantly reduced NM-induced apoptotic cell death to levels comparable to untreated corneas: NM 50 nM (32.5 ± 3.2% to 21.8 ± 4.7%), NM 100 nM (59.8 ± 3.8% to 24.1 ± 4.8%), and NM 200 nM (54.7 ± 7.8% to 23.2 ± 5.9%) (Figure 5D). These results suggest that MSC-CM decreased the level of NM-induced apoptosis.

**Figure 6:**
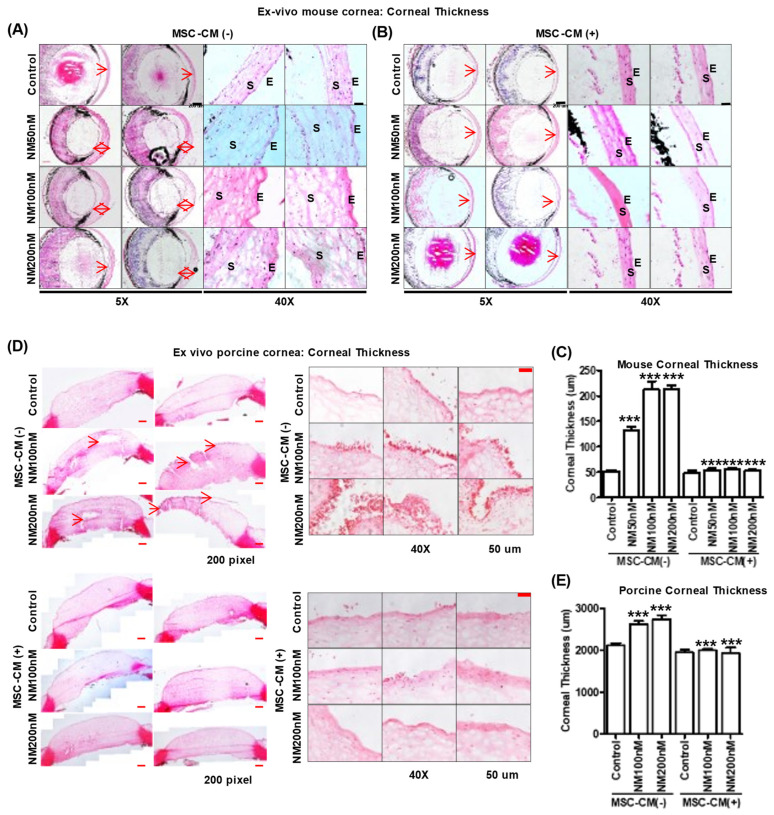
MSC-CM reverses NM-induced corneal epithelial damage in ex vivo mouse eye culture model. (**A**,**B**) Ex vivo mouse eye culture model: Representative images of H&E staining showing histology of mouse corneas after various doses of NM (50, 100, and 200 nM) in KSFM media for 2 h and washing twice with 1× PBS. After washing, NM-exposed mouse eyes were replaced with (**B**) or without (**A**) MSC-CM for 2 days and imaged using 5× and 40× magnification microscope. S: Stroma, E: Epithelium. (**C**) Graphs showing corneal thickness on mouse cornea area from Figure 6A,B. *** *p* < 0.001 vs. untreated control or NM treated group (MSC-CM-). (**D**) Ex vivo porcine eye culture model: porcine eyes were cultured in various doses of NM (50, 100, and 200 nM) in KSFM media 2 h and washed two times with 1X PBS. After washing, NM-exposed porcine eyes were replaced with or without MSC-CM for 3 days and imaged using 40× magnification microscope. (**E**) Porcine corneal thickness was measured from Figure 6D. *** *p* < 0.001 vs. untreated control or NM treated group (MSC-CM-).

Microscopic analysis of murine eyes showed corneal epithelial and stromal edema upon NM exposure compared with control (Control, 51.1 ± 9.5 μm; NM 50 nM, 131.3 ± 25.9 μm; NM 100 nM, 212.5 ± 44.3 μm; and NM 200 nM, 212.5 ± 23.1 μm) (Figure 6A). NM caused a loss of corneal endothelial cells and endothelial barrier integrity at Day 2 post-exposure. MSC-CM treatment after NM injury reduced epithelial swelling in mice corneas, NM 50 nM (131.3 ± 25.9 μm to 54.5 ± 9.3 μm), NM 100 nM (212.5 ± 44.3 μm to 55.0 ± 8.1 μm), and NM 200 nM (212.5 ± 23.1 μm to 53.1 ± 7.4 μm) (Figure 6B). Thus, NM exposure resulted in a 3–5-fold increase in corneal thickness depending on the concentration used while MSC-CM maintained corneal thickness similar to control levels (Figure 6C). Interestingly, increasing NM concentration dramatically increased damage to the central cornea, rather than the peripheral cornea or corneal–limbus area in mouse and porcine eyes (Figure 6). Moreover, with or without NM exposure, MSC-CM drives murine macrophages to express an M2 phenotype consistent with wound repair and anti-inflammatory effects (Supplementary Figure S4A,B). MSC-CM may therefore improve epithelial cell migration and mitigate macrophage-induced inflammation after NM injury. Both functions are consistent with improved wound healing.

To investigate the effects of MSC-CM on NM-exposed porcine eyes ex vivo, dissected porcine corneas were scratched and incubated in a 12-well plate for 2 h (Supplementary Figure S2). NM exposure increased whole corneal thickness by 24.2 ± 11.4% compared with the control eyes (Figure 6D). However, MSC-CM incubation reduced epithelial thickness at all concentrations of NM: NM 100 nM (2615.3 ± 239.3 μm to 1940.0 ± 188.954 μm) and NM 200 nM (2740.1 ± 284.0 μm to 1926.6 ± 296.6 μm) (Figure 6E).

**Figure 7:**
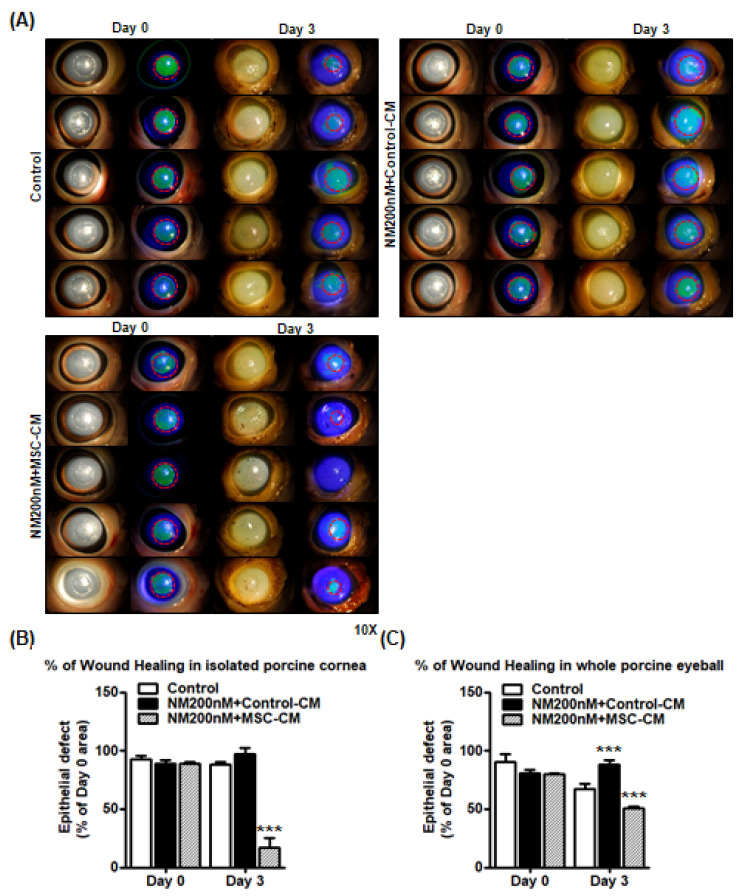
Wound healing effects of MSC-CM on NM injury in ex vivo porcine cornea culture model. (**A**) Ex vivo porcine cornea culture model for wound healing study: corneas were dissected from whole porcine eyes and a 6.5 mm wound was created on the porcine corneas. The 6.5 mm-wounded porcine corneas were cultured in various doses of NM 200 nM in KSFM media for 2 h and washed two times with 1X PBS. After washing, wounded porcine corneas were incubated with control (1:1 ratio of MEMa and 10% FBS/DMEM), NM 100 nM (in 1:1 ratio of time control media and 10% FBS/DMEM), and NM 200 nM (in 1:1 ratio of MSC-CM and 10% FBS/DMEM) for 3 days. A slit lamp was used to observe corneal staining (10× magnification) on Day 0 and Day 3. (**B**) Graph comparing epithelial defect area between groups (Control, NM 200 nM, and NM 200 nM + MSC-CM) at Day 0 to Day 3 (n = 5/group). *** *p* < 0.001 vs. NM 200 nM + Control CM (Day 3), (**C**) Graph comparing epithelial defect area between groups (Control, NM 200 nM, and NM 200 nM + MSC-CM) at Day 0 to Day 3 (n = 3/group) from Supplementary Figure S3. *** *p* < 0.001 vs. untreated control (Day 3). These results suggest that MSC-CM have the potential to enhance the healing process. Thus, MSC-CM reversed epithelial thickening and accelerated wound healing after NM injury both in vivo and ex vivo.

Next, we investigated the impact of MSC-CM on wound healing using an ex vivo porcine cornea model. We created an epithelial defect in porcine corneas and applied either control media, NM (200 nM), or NM with MSC-CM. NM exposure delayed wound healing in isolated porcine corneas (Figure 7A) while MSC-CM treatment accelerated wound healing with only 16.9 ± 18.1% of the epithelial defect present as compared with 88.1.3 ± 5.7% of epithelial defect present for the control (Figure 7B). As result, porcine whole eyeballs culture (ex vivo) showed more significant delayed wound healing after NM treatment on Day 3. Finally, we created a corneal dissection from porcine eye to be used for the following wound-healing experiment (Supplementary Figure S3). Similar to the previous experiment, MSC-CM treatment demonstrated significant acceleration of the wound healing process respectively in the whole porcine eyeballs after NM exposure (Figure 7C).

## 3. Discussion

Our goal was to investigate the effects of MSC-CM in NM-mediated delayed corneal wound healing, using in vitro, ex vivo, and in vivo models to establish our hypothesis that MSC-CM promotes corneal wound healing. The main findings of our studies were as follows: NM significantly induced (1) apoptotic cell death, (2) ROS generation, (3) cornea structural breakdown, and (4) delayed wound healing, whereas MSC-CM helped to restore mitochondrial function, resulting in increased cell survival after following NM exposure. MSC-CM treatment efficiently ameliorated NM-mediated delayed wound healing of the cornea. Based on these findings, we propose that MSC-CM has therapeutic potential to protect against chemical eye injury and it could be used in the development of targeted ocular therapies.

The toxic effects of NM exposure have been well established in previous studies. NM was shown to increase lipid profiles by activating the sphingomyelin–ceramide pathway [9]. NM exposure also caused DNA damage and severe systemic toxic effects in multiple murine organs, such as the eyes, liver, kidney, and spleen [2,3,40]. Our current findings revealed that NM decreased cell viability, increased intracellular ROS generation (Figure 1 and Figure 2), and decreased cellular ATP levels (Figure 1). However, MSC-CM incubation reversed many of NM-induced damages to cellular homeostasis. MSC-CM incubation also increased cell viability from 14.6–32.1% to 53.9%–76.7% after NM exposure in diverse cell line cultures (Figure 2A). It suggested that NM exposure induced changes in cell morphology as NM-exposed cells display the typical hallmarks of apoptosis, such as shrinking morphology. MSC-CM incubation recovered both cell viability and cell morphology (Figure 2). To search cornea-specific genes, such as metabolic gene expression in human cornea, the other investigators examined the gene expression profile of human corneal epithelium and found that the glucose transporter (Glut1), ATPase, and a-Enolase (glycolytic enzyme) were expressed in corneal epithelium and human corneal–limbal epithelial cells (HCLE) [41,42]. Glutamine catabolism (glutaminolysis) supports ATP synthesis in corneal endothelium function [43]. We have previously reported that inflammatory genes, IL-1β and TNF-α, are detected in corneal epithelial cells [44]. Therefore, we investigated the effect of MSC-CM on preventing pathologic responses in the cornea following chemical nitrogen mustard injury. In vitro data showed that NM exposure decreased metabolic gene expression, especially mitochondria-related genes (glycolysis: HK2, PFK, and PKM2; glutaminolysis: IDH2, IDH3G, and GLS2; ATP synthesis: ATP5S and ATP5D), while inflammatory genes (IL-1β, IL-10, and TNF-α) were increased (Figure 2B). Moreover, our data showed that NM decreased mitochondrial membrane potential which is the key indicator for mitochondrial activity in cells (Figure 3). As shown in Figure 3, NM exposure increased JC-1 monomers (low potential) and decreased JC-1 dimers (high potential) which indicated that MMP was reduced, resulting in a diminished level of cellular ATP (Figure 1C). Finally, MSC-CM therapy significantly restored the MMP following NM exposure. The reductions in mitochondrial membrane potential by NM exposure may contribute to the subsequent cellular ROS generation, which explains decreasing cellular ATP levels (Figure 1, Figure 3 and Figure 4). Charkoftaki G et al. analyzed the changes in lipid metabolites induced by NM and observed increases in sphingomyelins, ceramides, diacylglycerols, and platelet-activating factors [9]. They found that sphingomyelin metabolites were identified in nitrogen mustard-exposed corneal tissue extracts, although it was not clear which specific inhibitors blocked the potential sphingomyelin–ceramide pathway [9]. Several studies have demonstrated that NM-mediated activating MAPK/AktAP1 pathway led to the induction of inflammatory and proteolytic mediators in murine skin models, and NM-induced apoptosis is protected by vitamin D3 via activating VDR/Nrf2/Sirt3 pathway [38,39]. While we did not investigate the mechanisms underlying the protective effects of MSC-CM in our current study, considering our current focus on the therapeutic potential of mesenchymal stem cell-secreted factors on nitrogen mustard-induced corneal injury model, it will be of great interest to investigate the NM-involved mechanism in the future.

On the basis of these findings, we speculate that MSC-CM exerts protective effects on mitochondrial function and acts as an antioxidant as well. These results also suggest the possibility to change RNA splicing on metabolic enzyme genes leading to cellular malfunction and metabolic disorder. Therefore, future studies are needed to explore whether MSC-CM modulates DNA damage and mutagenesis induced by NM.

Our in vivo and ex vivo data similarly showed NM exposure delayed corneal wound healing whereas topical application of MSC-CM enhanced wound healing after NM exposure (Figure 5, Figure 7 and Supplementary Figure S3). Future studies are needed to determine the effects of MSC-CM after NM injury in naïve corneas (without wounding). NM exposure significantly increased epithelial edema in a dose-dependent manner, while the epithelial barrier integrity was preserved by MSC-CM incubation in both murine and porcine cornea models (Figure 6). NM exposure also induced loss of membrane integrity of surface epithelium and corneal stromal matrix, resulting in epithelial and stromal inflammation and apoptosis (Figure 6D and Figure 7).

However, MSC-CM therapy was able to reverse these effects and restore epithelial membrane integrity. MSCs and MSC-CM are widely accepted to use for anti-inflammatory and immunomodulatory effects [28,29]. Moreover, MSC-CM and cornea-derived MSC-CM modulated macrophages polarization [45,46,47]. As shown in Supplementary Figure S4, MSC-CM changes macrophage phenotype M1 to M2 type after NM exposure. These results suggest MSC-CM is an effective therapy for NM-mediated delayed corneal wound healing process in both in vivo and ex vivo models of the eye.

In this study, we have demonstrated that MSC-CM reverses NM-mediated delayed corneal wound healing process in corneal cells and tissues. Taken together, our results suggest that MSC-CM has potential therapeutic effects on mitochondrial stability, epithelial barrier function, and wound healing. MSC-CM may, therefore, be a candidate for development as a therapy to treat corneal epithelial wounds.

## 4. Materials and Methods

### 4.1. Cell Culture

Human corneal epithelial primary cells were isolated from human eyes following previously published methods [48,49]. Isolated human corneal epithelial primary cells and telomerase-immortalized human corneal–limbal epithelial cells (kindly provided by Ilene Gipson, Ph.D.) were cultured in keratinocyte serum-free medium (KSFM; Thermo, Waltham, MA, USA) with 0.05 mg/mL of Bovine Pituitary Extract and 5 ng/mL of Epidermal Growth Factor. Human corneal epithelial cell (HCEC) cultures were kindly provided by Illinois (Chicago, IL, USA) and Midwest Eye Banks (Ann Arbor, MI, USA) [49]. Human bone marrow (BM)-derived MSCs were kindly provided to author ARD’s research group at University of Illinois at Chicago (UIC) by Dr. Peiman Hematti’s group at University of Wisconsin Hospital and Clinics. BM-MSCs were cultured in alpha MEM media supplemented with 10% fetal bovine serum, 1x l-glutamine (Corning, NY, USA), and 1x NEAA (Corning, NY, USA) [50].

### 4.2. Preparation of Nitrogen Mustards and Treatment

Nitrogen mustard (NM; #122564, Sigma-Aldrich, St. Louis, MO, USA) was reconstituted in 1X PBS (25.9 M) and stored at −80 °C until the day of the experiment. For in vivo usage, a circular 2 mm-diameter epithelial wound was made over center of corneas, as previously reported [51]. After wound was created, mice were divided into following experimental groups: Control (PBS, n = 4); NM (100 nM, n = 3); and NM + MSC-CM topical application (n = 3) for 3 days. NM (100 nM, 10 μL) was applied to mouse eye for 30 min which was then washed out with 15 mL of 1X PBS and replaced with MSC-CM (10 μL) for 20–30 min. From second day, MSC-CM or PBS was applied two times a day for 3 days. The fluorescein staining was examined and imaged with a slit lamp for 3 days using 30× magnification and cobalt blue light with a yellow barrier filter. For in vitro studies, to determine the effect of nitrogen mustard on MSCs and corneal cells, the following conditions were used: control; NM; and NM+MSC-CM. Cornea cells were collected and processed to measure mRNA and protein levels on key metabolic enzyme genes.

### 4.3. Cell Viability

A concentration of 3 × 10^5^ cells/well with 6 wells per group were cultured in presence of various doses of nitrogen mustard (0.001–2 mM) for 2 h. Cells were rinsed with 1X PBS two times, then incubated with trypan blue (#T4049, Sigma-Aldrich Corp., St. Louis, MO, USA) for 10 min at room temperature. Trypan blue stained cells were observed under bright-field microscope (5X). Next, to monitor cell survival in vitro after NM 0.8 mM exposure, 1 × 10^6^ of HCEC, HCLE, HCEpC, and MSC were exposed to NM 0.8 mM in basal media (DMEM/F12 for HCEC and HCEpC, KSFM for HCLE, and MEMa for MSC) for 2 h and replaced with a 1:1 ratio of MSC-CM and basal media for 24 h after two washes.

### 4.4. MSC-CM

MSC-CM (conditioned medium of human bone marrow-derived stem cells) is generated as described previously [52]. The MSCs were characterized by flow cytometry, with more than 95% of cells positive for cell surface markers CD73 and CD90 and negative (less than 5%) for CD45, CD11b, HLA-DR, and CD14 at Passage 4 (Supplementary Figure S1A). Using a scratch assay to measure wound healing, we found that MSC-CM significantly enhanced wound healing at 12 h post-treatment compared with control media (Supplementary Figure S1B,C).

### 4.5. LDH Toxicity Assay

For LDH cytotoxicity assay, cells (HCEC, HCLE, HCEpC, and MSC) were seeded on 96-well plates at a concentration of 3 × 10^4^ cells per well, each in a medium. After 12 h incubation, cells were cultured in the presence of various doses of NM (0.001–2 mM) for 2 h, washed, and cultured for 24 h. After 24 h incubation, 50 μL of supernatant was mixed with 50 μL of reaction mix according to the manufacturer’s instructions (#C2030, ThermoFisher Scientific, Waltham, MA, USA) in a 96-well flat-bottom plate and incubated at room temperature for 30 min. Optical density absorbance at wavelengths (490–680 nm) was measured with a Cytation5 plate reader.

### 4.6. ROS Measurements

HCEC cells were plated in 4-well chamber slide at a concentration of 6 × 10^4^ for 12 h. After incubation, cells were cultured with a diverse range of NM for 2 h and washed and cultured with a 1:1 ratio of MSC-CM and basal media for 24 h. After 24 h, cells were treated with ROS/Superoxide Detection Mix reagent (#ab139476, Abcam, Cambridge, Massachusetts, UK) in a culture medium for 30 min. For confocal microscopy, cells were washed twice with PBS and overlayed with a cover slip. Cells were observed under a 20X Zeiss LSM 710 confocal microscope.

### 4.7. JC-1 Immunostaining

The mitochondrial membrane potential of HCEC was assessed with a JC-1 mitochondrial membrane potential assay kit (#ab113850, Abcam, Cambridge, Massachusetts, UK) according to the manufacturer’s instructions. Cells were seeded in 96-well dark plate at 2 × 10^5^ cells for 24 h. After 24 h, cells were treated with NM for 2 h and washed and cultured with a 1:1 ratio of MSC-CM and basal media for 24 h. For JC-1 assay, cells were incubated for 30 min at 37 °C with a 1 μM JC-1 solution or with the dilution buffer as the control followed by imaging in dilution buffer. For the fluorimetry experiment, we used an excitation wavelength of 475 nm and emission wavelengths of 530 ± 15 nm (monomer JC-1) and 590 ± 17.5 nm (dimer JC-1) which were measured with a Cytation5 plate reader. For the fluorescence microscopy experiment, confocal microscopy was performed on a 40X confocal microscope (Zeiss LSM 710 confocal microscope).

### 4.8. ATP Measurements

ATP levels were determined using the ATP Colorimetric/Fluorometric Assay Kit (#MAK190, Sigma-Aldrich, St. Louis, MO, USA). Cells (HCEC, HCLE, HCEpC, and MSC) were seeded in 96-well culture plates at a count of 2 × 10^4^ cells per well, incubated with nitrogen mustard (0.001–2 mM) for 2 h, washed, and incubated for 24 h. After 24 h incubation, 50 μL of sample was mixed with 50 μL of reaction mix in a 96-well flat-bottom plate and incubated at room temperature for 30 min. Optical density absorbance at wavelengths (570 nm) was measured with a Cytation5 plate reader.

### 4.9. Apoptosis Detection Kit

Apoptosis detection was determined using Apoptag in situ apoptosis detection kit (#S7110, Millipore, Bedford, MA, USA) according to the manufacturer’s instructions. Briefly, mouse cornea sections were post-fixed and added with Terminal deoxynucleotidyl transferase (TdT) enzyme in reaction buffer for 1 h. Sections were washed twice with 1X PBS and incubated with an anti-digoxigenin–fluorescein conjugate solution for 30 min at room temperature. Sections were washed with 1X PBS, and fluorescence was determined using a 20X Zeiss LSM 710 confocal microscope.

### 4.10. Porcine Cornea Model of Corneal Epithelial Wound Healing

Fresh intact porcine eyeballs were obtained from a certified abattoir (Park Packing Co. Inc., Chicago, IL, USA). The porcine corneas (PCs) were dissected and washed with 1X PBS containing 1% gentamicin, 1% penicillin, and 1% streptomycin [53]. A circular 2 mm diameter epithelial wound was made on center of the corneas as previously described [51].

### 4.11. Mouse Model of Corneal Epithelial Wound Healing

All animal experiments were conducted in compliance with the ARVO Statement for the Use of Animals in Ophthalmic and Vision Research. The protocol was approved by the Committee on the Ethics of Animal Experiments of University of Illinois at Chicago (UIC). In addition, the protocol was approved by the Biosafety Committee. Six-to-ten-week-old C57BL/6J mice were anesthetized with intraperitoneal injection of ketamine (100 mg/kg) and xylazine (5 mg/kg). After applying topical 0.5% proparacaine, a 2 mm-diameter epithelial wound was made on the center of cornea (Mil-Tec, Lehigh Acres, FL, USA) by using a burr (Algerbrush II, Allomed, Milwaukee, WI, USA) under a microscope (VistaVision, Champaign, IL, USA). Wounding of the cornea does result in temporary pain (1–2 days) which is partially controlled with analgesics. As indicated in the animal protocol, buprenorphine was used as analgesic. After the injury to the epithelium, mice were divided into two groups: saline control (n = 5) and MSC-CM (n = 5). For all the experiments, 10 μL of the topical treatment was applied to the right eye of each mouse twice a day.

### 4.12. Histology

Porcine eyes were placed in 50 mL tube and exposed to 5 mL of NM (100 nM and 200 nM) for 2 h. After 2 h treatment, porcine eyes were gently rinsed with 10 mL of 1X PBS and incubated with heat-inactivated MEMa media only or a 1:1 ratio of MSC-CM and heat-inactivated MEMa for 24 h. At the end of the experiment, for Hematoxylin and eosin (H&E) staining, porcine eyes were cut in half and embedded in OCT medium, cryo-sections at 10 µm and fixed in neutral buffered 10% formaldehyde (Sigma-Aldrich, St. Louis, MO, USA) for 20 min and followed the protocol as previously described [48,54,55]. Mouse eyes were placed in a 96-well plate and exposed to 200 µL of NM (100 nM and 200 nM) for 2 h. After 2 h treatment, mouse eyes were gently rinsed with 1 mL of 1X PBS and incubated with heat-inactivated MEMa media only or a 1:1 ratio of MSC-CM and heat-inactivated MEMa for 24 h. For Hematoxylin and eosin (H&E) staining, mouse eyes were embedded in OCT medium, cryo-sections at 10 µm and fixed in neutral buffered 10% formaldehyde (Sigma-Aldrich, St. Louis, MO, USA) for 20 min and followed the protocol as previously described [48,54,55].

### 4.13. In Vitro Scratch Assay

HCLE cells were seeded on 6-well plates at a concentration of 0.5 × 10^6^ cells per well, in KSFM complete with growth supplements. After 12 h incubation, cell monolayers were scratched using a sterile 200 μL pipette tip and washed twice with 1X PBS to remove floating cells. Then the heat-inactivated MEMa was added to control well and MSC-CM was added to experiment well for 2 h. The analysis of wound closure rate was evaluated every 2 h for 24 h using a spinning disc confocal microscope (Z1; Carl Zeiss Meditec, Jena, Germany), and the captured photographs (n = 5 wells per group) were analyzed using ImageJ software (National Institutes of Health, Bethesda, MD, USA, https://imagej.nih.gov/ij/, accessed on 10 March 2021) [56].

### 4.14. Macrophage Polarization and Flow Cytometry

J774.2 cells (mouse macrophage) were exposed in NM 200 nM for 2 h in 5% FBS/DMEM and washed twice with 1X PBS. After washing, cells were incubated with control-CM or MSC-CM for 36 h. Cells were stained with CD80 (#104713, BioLegend, San Diego, CA, USA), CD206 (#141703, BioLegend, San Diego, CA, USA), and 7-AAD (#420403, BioLegend, San Diego, CA, USA) for flow cytometry. For positive controls, 10 ng/mL LPS (InvivoGen, San Diego, CA, USA) and 1 ng/mL IFN-γ (#I4777, Sigma-Aldrich Corp., St. Louis, MO, USA) for 24 h were used to identify M1 macrophages, and 25 ng/mL IL-4 (#574302, BioLegend, San Diego, CA, USA) for 24 h was used for M2 macrophages identification. Gating strategy for flow cytometry: mouse macrophage cells were first gated on size and granularity followed by dye exclusion to identify live cells. Live cells were defined as M1-related macrophages (CD80high, CD206low) or M2-related macrophages (CD80low, CD206high) cells.

### 4.15. Real-Time PCR

HCEC cells were seeded in a 6-well plate at 2 × 10^5^ for 24 h. At 60–70% confluency, cells were exposed with NM (0.8 mM) for 2 h, after which they were gently rinsed two times with 1X PBS and cultured in heat-inactivated MEMa or 1:1 ratio of MSC-CM and heat-inactivated MEMa for 24 h. At the end of the experiment, total RNA was extracted from cell culture using an extraction reagent (TRIzol; Life Technologies, Carlsbad, CA, USA) according to the manufacturer’s protocol. The 2 μg of total RNA was used for cDNAs synthesis by High-Capacity cDNA Reverse Transcription Kit (Applied Biosystems, Carlsbad, CA, USA). The qPCR reactions were carried out in triplicate with FastStart Universal SYBER GREEN Master (Roche Applied Science, Mannheim, Germany) in a total volume of 20 μL using thermal cycling conditions of 10 min at 90  °C, 10 s at 95  °C, followed by 40 cycles of 95  °C for 15 s, and 60  °C for one minute. The gene expression of selected genes was evaluated by quantitative PCR (qPCR) methods (glycolysis: HK2, PFK, and PKM2; glutaminolysis: IDH2, IDH3G, and GLS2; ATP synthesis: ATP5S and ATP5D; and inflammatory genes: IL-1β, IL-10, and TNF-α). The value of the target gene was normalized, and the fold change was calculated using the 2^−ΔΔCt^ method (Table 1).

### 4.16. Statistical Analysis

Statistical calculations were conducted using GraphPad Prism software (GraphPad, La Jolla, CA, USA). Results are presented as the mean ± standard deviation (SD) of three independent experiments. P-values for differences were determined by double-sided nonparametric *t*-tests with GraphPad Prism software and Microsoft Excel. A *p* value <0.05 was considered statistically significant.

## Figures and Tables

**Table 1:** PCR primer sequences used for real-time PCR.

Gene	Forward	Reverse
*HK2*	CAAAGTGACAGTGGGTGTGG	GCCAGGTCCTTCACTGTCTC
*PFK*	GTACCTGGCGCTGGTATCTG	CCTCTCACACATGAAGTTCTCC
*PKM2*	AAGGGTGTGAACCTTCCTGG	GCTCGACCCCAAACTTCAGA
*IDH2*	CGCCACTATGCCGACAAAAG	ACTGCCAGATAATACGGGTCA
*IDH3G*	ACATCGAAACCAACCATAACCTG	GGCTCTTACAGTGGATGACGTT
*GLS2*	TGCCTATAGTGGCGATGTCTCA	GTTCCATATCCATGGCTGACAA
*ATP5S*	AGCAGTTGTGTGGCGTAAAGA	CTGATGCGATCATAATCCACCTT
*ATP5D*	ACTCTTCGGTGCAGTTGTTGG	GCCTCGATTCGGATCTGGAT
*IL-1β*	CTGCTCTGGGATTCTCTTCAG	ATCTTCCTCAGCTTGTCCATG
*IL-10*	TCTCCGAGATGCCTTCAGCAGA	TCAGACAAGGCTTGGCAACCCA
*TNF-α*	CTTCTCCTTCCTGATCGTGG	GCTGGTTATCTCTCAGCTCCA
*GAPDH*	GCCAAAAGGGTCATCATCTC	GTAGAGGCAGGGATGATGTTC

## Data Availability

Any data or material that support the findings of this study can be made available by the corresponding author upon request.

## Supplementary Materials

The supporting information can be downloaded at: https://www.mdpi.com/article/10.3390/ijms231911510/s1.
