# Supplementary material for: Therapeutic Potential of Mesenchymal Stem Cell-Secreted Factors on Delay in Corneal Wound Healing by Nitrogen Mustard
Source: Int J Mol Sci. 2022 Sep 29;23(19):11510. doi: 10.3390/ijms231911510 (PMC9570439; doi:10.3390/ijms231911510)
Supplement: Supplementary file 1 [file ijms-23-11510-s001.zip › ijms-1858283-supplementary.pdf]

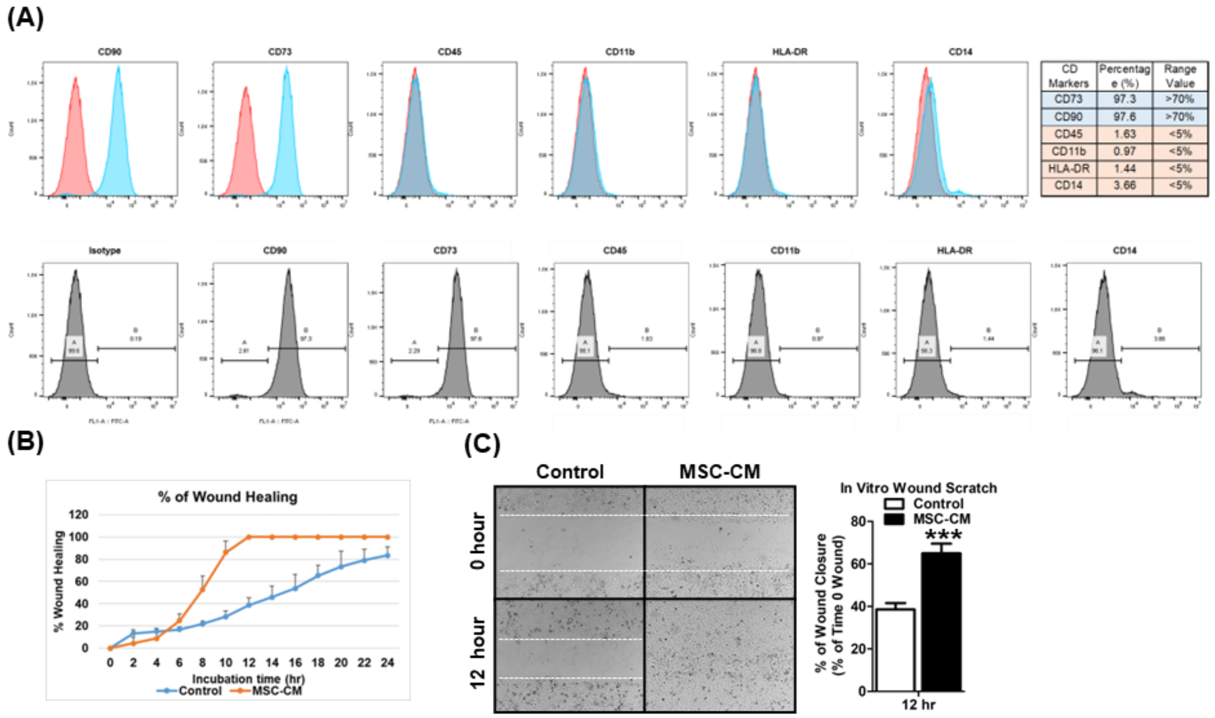

Supplementary Figure S1. Surface marker characterization of human bone marrow-derived MSCs by fluorescent activated cell sorting. Passage 3, BM-MSC cells (1 x 10^6) from Rooster cells were grown in Rooster Bio XF media for 2-3 days until they became 70-80% confluent. After washing twice with 1X PBS, the media was changed to 10 mL of Rooster-Collect media only and incubated for 72 h in a T75 flask. At the end of the experiments, cell number will be approximately 3-3.5 million in total. They were then collected in 10 mL of Rooster-Collect media after 72 h incubation and centrifuged at 500g for 5 min for cell-free CM. Human corneal-limbal epithelial cells were grown in a 6-well plate with KSFM media. When the cells became 100 % confluent, a vertical scratch was made in the middle of wells and incubated with 50% KSFM media + 50% MSC-CM for the experimental group or 50% KSFM media + 50% MEMa media for control group. (A) Characterization of Bone Marrow Multipotential Stromal Cells. (B) MSC-CM incubation enhances wound healing compared to the control group in a time series. (C) The area of the wound is calculated by imaging software. Wound healing ratio was compared with control at 12 h incubation time point.

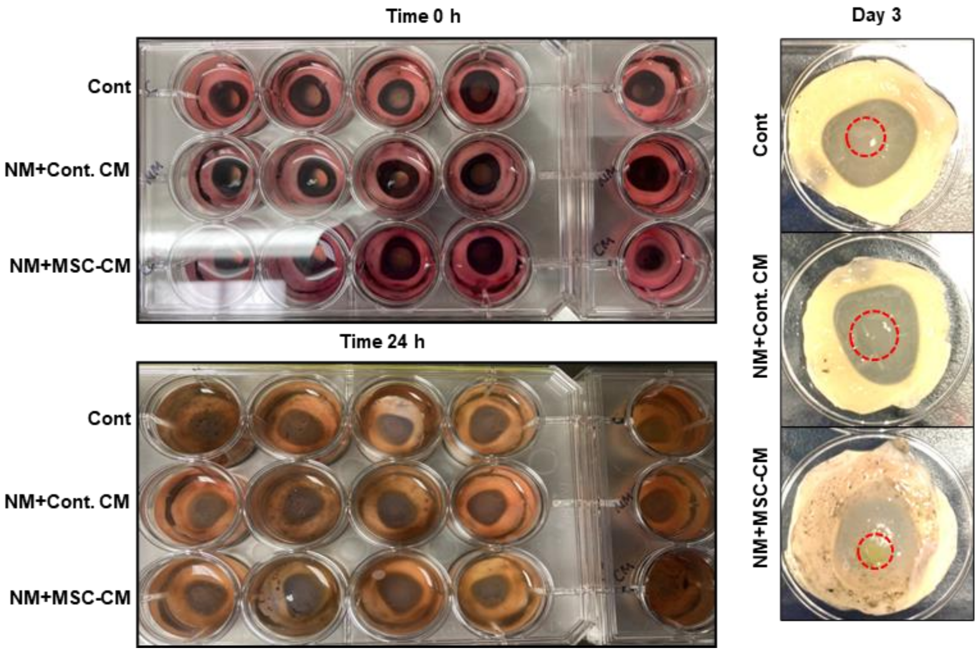

Supplementary Figure S2. Dissected porcine corneas and wound healing image. Corneas were dissected from whole porcine eyes and used for the following wound-healing experiment. At the time 0 h, dissected corneas were incubated under following conditions (Control, NM + control media, and NM + MSC-CM, 24 h).

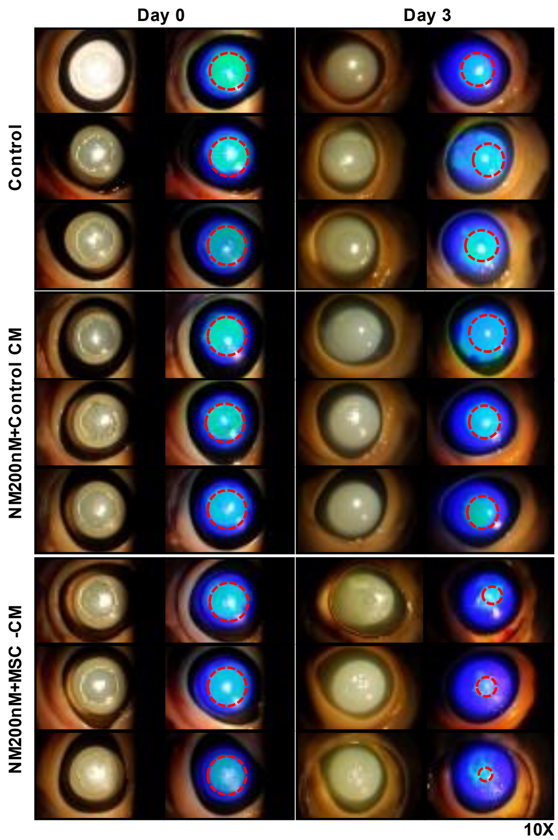

Supplementary Figure S3. Wound healing effect of MSCs-secreted factors on NM injury in *ex vivo* whole porcine eye culture model. *Ex vivo* porcine eye culture model for wound healing study: 6.5mm-wounded porcine eyes were cultured in various doses of NM 200 nM in KSFM media for 2 h and washed two times with 1X PBS. After washing, wounded porcine eyes were incubated with control (1:1 ratio of MEMa and 10% FBS/DMEM), NM 100 nM (in 1:1 ratio of time control media and 10% FBS/DMEM), NM 200 nM (in 1:1 ratio of MSC-CM and 10% FBS/DMEM) for 3 days. A slit lamp was used to observe corneal staining (10X magnification) on day 0 and day 3 (n=3/group).

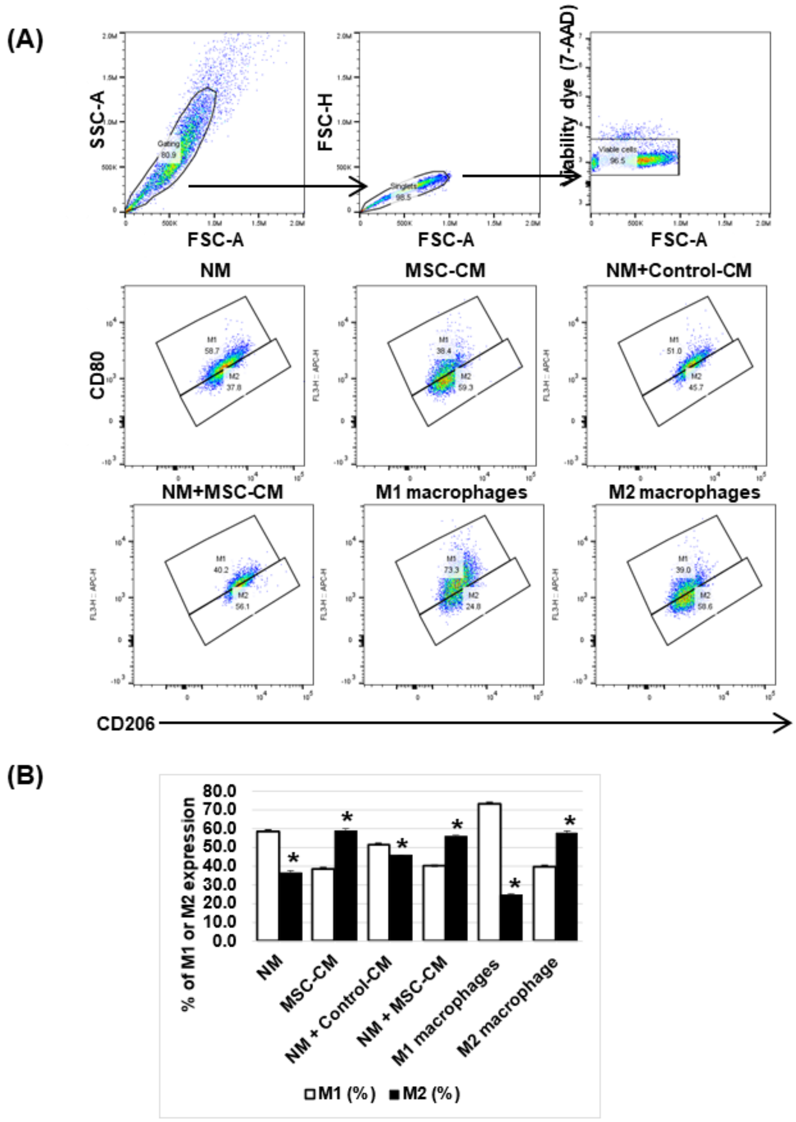

Supplementary Figure S4. MSC-CM changes surface expression levels of M1 and M2 murine macrophages after NM exposure. (A) Mouse macrophage cells were exposed to NM 200 nM for 2 h and washed two times with 1X PBS. After washing, mouse macrophage cells were incubated with control-CM or MSC-CM for 36 hr. After CM incubation, cells were stained with CD80 (BioLegend, #104713), CD206 (BioLegend, #141703), 7-AAD (Sigma, #S8032) for flow cytometry. (B) Graph comparing the % of M1 and M2 macrophage phenotype change in Control, NM 200 nM, and NM 200 nM + MSC-CM. *$P < 0.001$ vs. M1 (%).
